# Some Accidents and Complications Incident and Subsequent to Abdominal and Pelvic Operations1Read at the annual meeting for the American Association of Obstetricians and Gynecologists, at Cincinnati, O., Sept. 18, 1889.

**Published:** 1889-11

**Authors:** Joseph Hoffman

**Affiliations:** 126 West Diamond street; Philadelphia, Pa.


### SOME ACCIDENTS AND COMPLICATIONS INCIDENT AND SUBSEQUENT TO ABDOMINAL AND PELVIC OPERATIONS.[1]

By JOSEPH HOFFMAN, M. D., Philadelphia, Pa.

THE time is come to acknowledge that the successes of abdominal and pelvic surgery are bounded and measured by the ability to master their complications. The field of gynecic surgery to-day is plowed and harrowed by as restless and motley a throng as ever surged upon the Pacific slope for gold. Too often their zeal is not for earnest work, but for local renown. They would build a tower to make themselves a name. The end must be confusion and complaint. These are troublesome times for abdominal surgery and surgeons. They have their way to make and sustain both against the inherent difficulties of their work, and against the failures of those who insist on "opening the abdomen because it is easy." For this condition of matters abdominal surgeons are, for the most part, to blame, by reason of ill-advised expression as to the difficulty of the work. When Mr. Tait says in effect that the removal of the entire pregnant uterus is an easy matter that

1. Read at the annual meeting for the American Association of Obstetricians and Gynecologists, at Cincinnati, O., Sept. 18, 1889.

every country surgeon should be ready to perform, it is unnecessary further to explain this point. It is easy with special training in the technique of abdominal surgery, but most difficult without this. So it is in all the varieties of pelvic and abdominal work. The removal of an organ is often the least part of the operation. The attention to the minute details that alone can bring success to the operation, is only attained by long and careful training. It has been asserted that the operation is nothing, that the after-treatment is the most important consideration. My own experience is diametrically opposed to this assertion. Prolonged treatment after abdominal section should be nearly or quite as rare as repeated operation upon the same patient. In a majority of instances, the man who operates two or three times upon the same patient for the same allied affection, has operated carelessly or inefficiently at first, and will probably do the same at last. This is the man who will find prolonged after-treatment necessary, and who probably considers the operation itself of secondary importance. Let us start, then, with the idea that the operation is a serious undertaking, to be seriously performed, after serious consideration; we are to remember, at the same time, that what appears the simplest may eventuate in the most complicated condition, and that herein the necessity for careful preparation in all that belongs to abdominal or pelvic surgery is the *sine qua non* of assured success. A knowledge of what may happen is the best protection against misfortune, and the factor of the extreme simplicity in the work of the best surgeons.

The accidents and complications must, of course, vary according to the organ dealt with or the nature of the operation. In the presence of adhesions we may have hemorrhage or intestinal rupture. These are most apt to occur in long-standing pelvic inflammations, and in extra-uterine pregnancy. Case XXVI. of my series well illustrates both these complications. Extrauterine pregnancy with pyosalpinx produced a complication as hard to describe as to recognize. The intestine was torn off too low to bring the ends out for artificial anus, while the hemorrhage was such that the pelvis was packed to control it. The packing remained for sixty hours, when it was removed without return of the bleeding. The intestine was carefully sutured deep down in the pelvis, and, strangely enough, in the face of the extreme difficulty of this procedure, the woman recovered without fecal or other fistula. The case brings out the necessity of exact knowledge of intestinal suturing, and the importance of packing in pelvic hemorrhage. Half-way measures in these and similar cases must always result disastrously. In gonorrheal disease of the tubes and ovaries where the disease was of long standing, there is frequently found such a degeneration of the tissues that it is impossible to make an ideal stump or button for safe tying. Luckily, here, as in other degenerations, the blood-vessels are the last to yield their integrity; and when they are intact, though the stump is not ideal, the hemorrhage can be controlled. Case XXII. is a typical illustration of this condition in its extremity. The tissues were so rotten that the ligature cut through broad ligament, vessels, and all. The cornu of the uterus was curetted and the ligature applied directly to the uterine tissue. The degeneration of tissues, such as is here referred to, is not uncommon in all cases of pyosalpinx, whatever their origin. The importance of curetting away the diseased parts cannot be too strongly urged as the most important step outside the line of ordinary procedure. A complication of pelvic surgery, always vanquishing the neophyte and taxing the ingenuity of the experienced, is the tying off of rudimentary appendages, (Cases XXIV. and XXVII.,) at the bottom of the pelvis. Nothing is easier than to tie off the normally developed tube and ovary; but when these lie at the bottom of the pelvis, almost entirely bound down in its peritoneum, to tie them off—done, as it must be, by the ends of the fingers, through an incision two inches long—requires an amount of manual dexterity only attained by much and patient endeavor. Now, as the application of the ligature may greatly complicate and delay the operation, so slipping may give rise to greater trouble. After careful tying, the greatest safeguard against slipping is the leaving of a generous button. There is no necessity for complicated knots with fancy names. The ordinary surgeon's knot, applied as it should be, will never fail to hold.

The more deliberately the tying is done, thereby affording brief time for shrinkage, the less likely is oozing to occur. The smoother the ligature, the better it will tie. For this reason I prefer cabled to braided silk.

In deep pelvic surgery there is constant danger of wounding the ureters. This, in the present status of our knowledge, involves a most serious procedure—the removal of the corresponding kidney. There is open here a wide field for vivisection experiment, to ascertain with what anatomical and physiological success the ureters can be grafted into the intestine. If this can be successfully done in the human being, another important stride in surgery will have been taken.

The presence of dense adhesions, especially visceral, and the consequent hemorrhage, for a long time baffled the best attempts of the surgeon. Clots and débris of every description found their way and remained in every nook and cranny of the peritoneal cavity, from diaphragm to pelvic floor. These could not fail to do infinite harm, and the drainage-tube was entirely inadequate to remove them. This is, indeed, no easy task. As ordinarily attempted with the syringe, it is often unsuccessful, because the current of water is not strong enough to find egress with sufficient momentum to bring away the particles—often, indeed, masses—of lymph and clot. At the present, the gravity tube gives the best method of thorough cleansing. The water is poured into a funnel elevated according to the effect desired, while the other end, fitted with a nozzle, is directed all over the peritoneal cavity, under the broad ligaments, mesentery, liver, spleen, and, in fact, everywhere that clot can lurk. The free drenching or flooding of the abdominal cavity need not be feared; the heat is an important desideratum in relieving shock. I have constantly employed it, even where drainage was afterward found unnecessary, and have yet to find a single bad result. This has also been the experience of my friends who operate more frequently than myself. Operation with existing pregnancy is a complication not much referred to. It is, no doubt, frequently met, and just as frequently concealed. In one such case, I have operated to save life. The woman did not think herself pregnant, was suffering from symptoms of peritonitis, belly tympanitic and tender, had high fever, and I felt, as I delayed operation a day or two, while I tried, without success, to relieve her with salines and external applications, that I was treading upon dangerous ground. Operation revealed general pelvic peritonitis, with pus in the tubes. The uterus was enlarged, which previously was in doubt, owing to the size and distension of her belly. Three days later, the patient miscarried, her pain, in the meanwhile, being controlled with morphine and atropine. She recovered, and is now among the best of my results. It is evident that, in cases such as this, the tying of the ligatures is an important consideration, owing to the increased strain put upon them, both by uterine contraction and abdominal tension. I believe, in these cases, morphine or other anodyne is of distinct advantage. Here I met also another complication. The unrest of the patient, before I put her under anodynes, was so great and violent that the drainage-tube was displaced. I felt there was need of it, owing to the extreme inflammation, and tried to reintroduce it. In this I failed, and had to remove it. For a day or two, the symptoms were urgent, and I made preparation to reopen and drain, but, happily, under free purgation, she became comfortable, and this was unnecessary. This was Case XIV.

The careful disinfection of suspicious tubes, before the stumps are allowed to drop, is a step, I am convinced, should never be omitted. I believe that a failure to do this in one of my cases, apparently very simple, was the cause of peritonitis and death. A previously induced abortion, of which I was ignorant at the time, was, I believe, the immediate cause of the sepsis.

Among the immediate post-operative complications, peritonitis was, until lately, reckoned as most likely and most fatal, excepting, perhaps, hemorrhage. This is, in great measure, no longer true. Peritonitis must be distinguished from simple serous exudation. This latter is the result of simple exudation from wounded capillaries, broken-down adhesions, or even, perhaps, from the sometimes necessarily rough handling of the peritoneum. In the absence of specific cause, this, if drained away, does not cause peritonitis. Hence the philosophy of draining, both to remove possible pabulum for infection, and to take away the serum which hygroscopically attracts fluids by its presence. It is a physical law that moisture attracts moisture, and in the abdomen there is no exception to the rule. Where chemical antisepsis is used, there is greater liability to this serous exudation. There is thus also greater danger of post-operative visceral adhesions. This is illustrated in Case VIII. The patient had before been operated upon for uterine fibroid. A drainage-tube was put under the care of her husband, and the abdominal cavity washed out with carbolic solution, of whose strength I am ignorant. When the abdomen was opened, the intestines were found matted together and out of all natural position.

The transverse colon was adherent to the old incision. The remainder of the colon, almost throughout its whole length, was adherent to the other viscera. To free them, nearly all the intestines had to be handled. No other cause for this condition need be sought than the free use of carbolic acid.

That the saline or calomel treatment of serous exudation is of undoubted value after operation, does not admit of dispute. It is just as clear that it excels all other remedies. This is at least true so far as abdominal surgeons are concerned. It has now been so long insisted upon that it is an old story, and were it not that general practitioners fail to appreciate its value, for the most part, its mention here would be superfluous. Case XXIX. in my list is indisputable evidence of the value of this treatment. The condition of the woman was such that after all preparation for operation had been made, by the advice of Dr. Joseph Price, I postponed operative interference. The woman's pulse was thready and frequent, her belly tympanitic and tender, with every evidence of peritonitis. I at once put her on the mercurial treatment, administering a grain of calomel every hour, until twenty-four grains had been taken, stimulating her at the same time. The result was a complete subsidence of the acute symptoms and a vast improvement in the pulse-rate, and after a few days I operated. The peritonitis was general, and of a virulent type, the tubes being full of pus. After rather a tedious convalescence, she recovered. Her only inconvenience now is a fistula, through which a ligature has lately escaped. This, I think, is owing more to her condition at the time of operation. Her bodily condition was such that none of the stitches held, the abdominal incision falling apart, and the entire wound had to heal by granulation.

This brings us in sequence to the consideration of fistulas. The drainage-tube is often charged with producing this complication. I can readily understand how this may be the case, especially in the causation of fecal fistula, where the tube is allowed for a long time to make harmful pressure upon the intestine. I believe, however, that, in a great majority of cases, the fistula has its origin in the ligature applied to the pedicle, or in a systemic vice. One of the most annoying fistulas I ever encountered occurred in Case XXII., where no drainage-tube had been used at all. In the course of time it healed, and the incision is now perfect. In another case, just now closing, the operation was for the removal of the appendages, after a puerile attempt by another to hold the uterus in position by stitching it against the abdominal wall, while the ovaries were bound down by adhesions. The persistence of the fistula here, Case XXIII., may be due to incision through the cicatricial tissue. I am certain I can give no other reason. For fecal fistula, except where the bowel is extensively injured or deeply torn, without chance of suture, I think there is little excuse. Carelessness in examining the intestine after extensive breaking up of adhesions, or careless tying and omental inclusion, are perhaps generally at the bottom of the calamity. A tedious closing of an incision, I do not regard as a misfortune. It keeps the patient longer on her back, thus favoring more solid cicatrization, and diminishing the chance of that other reproach of abdominal surgery—hernia. This accident I have never had to occur primarily. It exists in two of my cases, but it was present at the time of operation. In fact, in Case II., the operation was done to relieve strangulation, after the woman had vomited immense quantities of fecal matter. Her belly was, and is, enormous, and though she has never had trouble since her operation, a large hernia still exists. Certain it is, that no drainage-tube was the cause in this case. In a discussion of this subject, Mr. Skene Keith, some time since, reported the accident occurring in some cases, all of which had a short incision. He argued that, apparently, the short incision favors the complication, because of all the abdominal tensions being directed to a small, weak cicatrix. The fallacy lies in supposing that a long weak line will be stronger than a short weak line of incision. If the line is short, the mass of intestine able directly to impinge upon it, is correspondingly less. I cannot see that the law of hydrostatic pressure applies mathematically in the abdomen. My own experience contradicts this presumption, as does also that of many of my fellow-operators. From my own observation, I entirely believe in the correctness of Price's view, that three causes contribute most hernias, to-wit: imperfect suturing, in which the abdominal tissues are not brought into proper juxtaposition; too early rising after operation; and the too early abandonment of the abdominal supporter. To these may be added as a cause, the very long incision.

Reoperation brings with it its own peculiar complications. Usually these are in the form of adhesions. This has been the condition in three of my cases, IX., XX., and XXIV. In all of them, adhesions were present between the intestines and the abdominal cicatrix. This evil is best avoided by a careful bringing down of the omentum in front of the intestines and under the incision, before closing it. This is a point too often neglected. A complication, the cause of which is, for the most part, at the present only guessed at, and alike worrysome to both patient and surgeon, is post-operative hemorrhage. In most cases the patient, after the removal of the tubes and ovaries, is encouraged to believe that her hemorrhages, whether catamenial or otherwise, will cease. Everything goes on according to promise, until suddenly, from three to six months after the operation, hemorrhage occurs, to the fright of the patient and the disgust of the surgeon. This has happened in a number of my cases. The cause is still a matter of conjecture, hyperplasia of the intra-uterine mucosa being apparently a factor in most cases. Why this should supervene is not clear.

Curetting, hot douches, and iodine are efficacious in some cases, while in others the bleeding continues at various intervals. A rational explanation of this condition is much to be desired. Under this head I do not mean to include hemorrhage from malignant degeneration.

With this brief consideration of some of the difficulties of abdominal section, it would appear clear that operation is not a matter of routine, but rather of inspiration. Each separate case is a study of itself, and its complications a separate study. Successful operation means first a study of details; after this, careful observation and a gradual comprehension of principles. No operator of the present day has a right to start out with a mortality of ten years ago. No man has a right to record a death because he does not know how to tie a ligature. Operators are required, but only those are fitted to operate who are content to study principles and learn to apply details under efficient direction.

Appended is a table of all my cases to date, a study of the individual operations of which has afforded the subject-matter of this paper:

| No. | Date. | Diseases. | Result. | Drainage. |
|---|---|---|---|---|
| 1 | July, 1886 | Cirrhotic tubes and ovaries . . . . . . | Recovery. | |
| 2 | Sept. 4, 1887 | Strangulated ventral hernia . . . . . . | " | |
| 3 | May 15, " | Suppurating appendix; hematoma . . . | " | Yes. |
| 4 | May 21, 1888 | Ovarian and tubal adhesions . . . . . | " | |
| 5 | June 8, " | Septic salpingitis . . . . . . . . . . | Death. | No. |
| 6 | June 30, " | Double pyosalpinx . . . . . . . . . . | Recovery. | Yes. |
| 7 | July 22, " | General adhesions of tubes and ovaries | " | " |
| 8 | Aug. 3, " | Ovarian, tubal, abdominal, and pelvic adhesions . . . . . . . . . . | " | " |
| 9 | Aug. 14, " | Occluded tubes . . . . . . . . . . | " | |
| 10 | Aug. 16, " | Ovarian and tubal inflammation . . . . | " | |
| 11 | Sept. 23, " | Adherent tubes and ovaries . . . . . | " | |
| 12 | Sept. 23, " | Double pyosalpinx . . . . . . . . . . | " | Yes. |
| 13 | Sept. 27, " | Papilloma; exploratory . . . . . . . | " | " |
| 14 | Sept. 30, " | Pyosalpinx; pregnancy . . . . . . . | " | " |
| 15 | Oct. 17, " | Double pyosalpinx; ovarian . . . . . | Death. | " |
| 16 | Oct. 24, " | Omental hernia . . . . . . . . . . | Recovery. | |
| 17 | Oct. 25, " | Exploratory . . . . . . . . . . . . . | " | |
| 18 | Nov. 5, " | Salpingitis and ovaritis . . . . . . . | " | |
| 19 | Nov. 9, " | Ovarian cyst; salpingitis . . . . . . | " | |
| 20 | Nov. 25, " | Adhesions to old incision . . . . . . | " | |
| 21 | Dec. 8, " | Salpingitis; ovarian hypertrophy . . | " | |
| 22 | Dec. 18, " | Double pyosalpinx . . . . . . . . . . | " | Yes. |
| 23 | Feb. 17, 1889 | Adhesions, salpingitis, ovaritis . . . . | " | " |
| 24 | Feb. 23, " | Dermoid, infantile uterus . . . . . . | " | |
| 25 | March 6, " | Cyst and salpingitis . . . . . . . . . . | " | |
| 26 | March 17, " | Extra-uterine pregnancy and pyosalpinx | " | Yes. |
| 27 | March 19, " | Ovaritis; adhesive rudimentary tubes and ovaries . . . . . . . . . . | " | |
| 28 | March 29, " | Chronic salpingitis . . . . . . . . | " | |
| 29 | April 5, " | Double pyosalpinx . . . . . . . . . | " | Yes. |
| 30 | April 12, " | Dermoid cyst . . . . . . . . . . . | " | " |
| 31 | April 19, " | Chronic salpingitis . . . . . . . . . | " | |
| 32 | May 19, " | Ovarian cyst . . . . . . . . . . . . | " | |
| 33 | June 1, " | Chronic salpingitis . . . . . . . . . | " | |

Examination of the figures shows that the drainage-tube was used in thirteen cases, or in about forty per cent. of all operations. Had it been used in another case, I believe I would have one fewer death to record. No hernias have occurred.[1] All the patients operated upon are alive but four, two having died since operation. The others are all able to attend to their household duties, save one or two. No patient is worse for her operation, unless I am ignorantly in error.

1. Since writing the above, I have discovered one hernia.

The number of deaths is two; one patient dying of sepsis the tenth day after the operation; the other never rallied, and died of shock. In the last twenty-eight operations one death occurred. In the last eighteen cases all recovered.

The complication that has been most annoying is post-operative metrorrhagia.

126 WEST DIAMOND STREET.

